# A155 STOOL-BASED PROTEIN SIGNATURES FOR NON-INVASIVE ACCURATE DIAGNOSIS AND SUBTYPING OF INFLAMMATORY BOWEL DISEASE THROUGH HIGH-THROUGHPUT PROTEOMICS AND MACHINE LEARNING APPROACHES

**DOI:** 10.1093/jcag/gwae059.155

**Published:** 2025-02-10

**Authors:** E Shajari, d gagne, M Malick, P Roy, M Delisle, M Brunet, F Boisvert, J Beaulieu

**Affiliations:** Department of Immunology and Cell Biology, Faculty of Medicine and Health Sciences, Université de Sherbrooke, Sherbrooke, QC, Canada; Department of Immunology and Cell Biology, Faculty of Medicine and Health Sciences, Université de Sherbrooke, Sherbrooke, QC, Canada; Department of Immunology and Cell Biology, Faculty of Medicine and Health Sciences, Université de Sherbrooke, Sherbrooke, QC, Canada; Department of Immunology and Cell Biology, Faculty of Medicine and Health Sciences, Université de Sherbrooke, Sherbrooke, QC, Canada; Department of Medicine, Faculty of Medicine and Health Sciences, Université de Sherbrooke, Sherbrooke, QC, Canada; Department of Pediatrics, Faculty of Medicine and Health Sciences, Université de Sherbrooke, Sherbrooke, QC, Canada; Department of Immunology and Cell Biology, Faculty of Medicine and Health Sciences, Université de Sherbrooke, Sherbrooke, QC, Canada; Department of Immunology and Cell Biology, Faculty of Medicine and Health Sciences, Université de Sherbrooke, Sherbrooke, QC, Canada

## Abstract

**Background:**

Accurate diagnosis of inflammatory bowel disease (IBD) is essential to distinguish it from other conditions with similar symptoms and to identify whether it’s ulcerative colitis (UC) or Crohn’s disease (CD), ensuring appropriate treatment and management. While colonoscopy and biopsy are the current gold standards, they are invasive, costly, and poorly accepted by asymptomatic patients. Fecal biomarkers like calprotectin are commonly used but lack the specificity and lack of ability to differentiate between CD and UC, highlighting the need for more precise, non-invasive diagnostic methods.

**Aims:**

This study aims to develop a stool-based protein biomarker panel capable of accurately distinguishing IBD from IBD-mimicking conditions. It also seeks to classify the subtypes, CD and UC, by using high-throughput Data-Independent Acquisition mass spectrometry (DIA-MS) to identify precise biomarker signatures from complex stool samples, combined with advanced machine learning techniques for developing predictive model.

**Methods:**

Stool samples were collected from 46 active-CD patients, 23 active-UC patients, and 53 patients with conditions presenting similar symptoms. Using DIA-MS, we analyzed the stool proteome, identifying and quantifying proteins. Data processing procedures were carefully optimized to establish a robust analytical pipeline. The samples were then split into training and testing groups. Feature selection algorithms were applied to the training group to identify proteins that significantly differed between the groups. Six machine learning algorithms (kNN, Naive Bayes, eXBoost, Random Forest, SVM, and glmnet) were subsequently evaluated to determine the best-performing classifier.

**Results:**

Signature 1, consisting of 7 proteins for diagnosing true symptomatic IBD cases, and Signature 2, comprising 8 proteins for distinguishing between CD and UC, were developed using the best-performing classifier on the training dataset. Signature 1 achieved an AUC of 0.98, while Signature 2 reached an AUC of 0.96. For validation, the final predictive model was applied to a set of unseen samples. Impressively, the model demonstrated an AUC of 0.96 for both classifications, confirming its robustness and ability to generalize effectively to new samples.

**Conclusions:**

In conclusion, this study illustrates the effectiveness of utilizing stool proteome obtained through DIA-MS in accurately diagnosing and subtyping active IBD. Further future validation on a larger cohort using targeted MRM mass spectrometry would be served to establish the clinical utility of this approach.

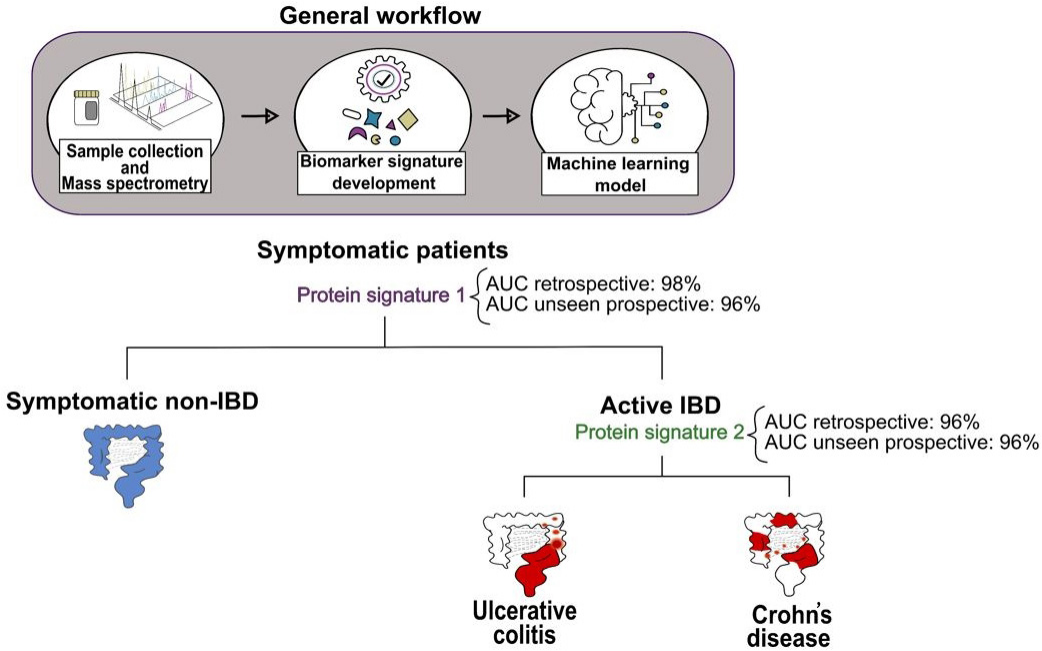

**Funding Agencies:**

CCCFaculty of Medicine and Health Science of University of Sherbrooke

